# Thermochemical Stability and Friction Properties of Soft Organosilica Networks for Solid Lubrication

**DOI:** 10.3390/ma11020180

**Published:** 2018-01-24

**Authors:** Pablo Gonzalez Rodriguez, A. Petra Dral, Karin J. H. van den Nieuwenhuijzen, Walter Lette, Dik J. Schipper, Johan E. ten Elshof

**Affiliations:** 1 Materials Innovation Institute (M2i), Elektronicaweg 25, 2628 XG Delft, The Netherlands; pagonro@gmail.com; 2 Inorganic Materials Science, MESA+ Institute for Nanotechnology, University of Twente, P.O. Box 217, 7500 AE Enschede, The Netherlands; a.p.dral@utwente.nl (A.P.D.); k.j.h.vandennieuwenhuijzen@utwente.nl (K.J.H.v.d.N.); 3 Surface Technology and Tribology, Faculty of Engineering Technology, University of Twente, P.O. Box 217, 7500 AE Enschede, The Netherlands; w.lette@utwente.nl (W.L.); d.j.schipper@utwente.nl (D.J.S.)

**Keywords:** lubricants, silica, hybrids, coefficient of friction, oxides

## Abstract

In view of their possible application as high temperature solid lubricants, the tribological and thermochemical properties of several organosilica networks were investigated over a range of temperatures between 25 and 580 °C. Organosilica networks, obtained from monomers with terminal and bridging organic groups, were synthesized by a sol-gel process. The influence of carbon content, crosslink density, rotational freedom of incorporated hydrocarbon groups, and network connectivity on the high temperature friction properties of the polymer was studied for condensed materials from silicon alkoxide precursors with terminating organic groups, i.e., methyltrimethoxysilane, propyltrimethoxysilane, diisopropyldimethoxysilane, cyclohexyltrimethoxysilane, phenyltrimethoxysilane and 4-biphenylyltriethoxysilane networks, as well as precursors with organic bridging groups between Si centers, i.e., 1,4-bis(triethoxysilyl)benzene and 4,4′-bis(triethoxysilyl)-1,1′-biphenyl. Pin-on-disc measurements were performed using all selected solid lubricants. It was found that materials obtained from phenyltrimethoxysilane and cyclohexyltrimethoxysilane precursors showed softening above 120 °C and performed best in terms of friction reduction, reaching friction coefficients as low as 0.01. This value is lower than that of graphite films (0.050 ± 0.005), a common bench mark for solid lubricants.

## 1. Introduction

**Scheme 1:**
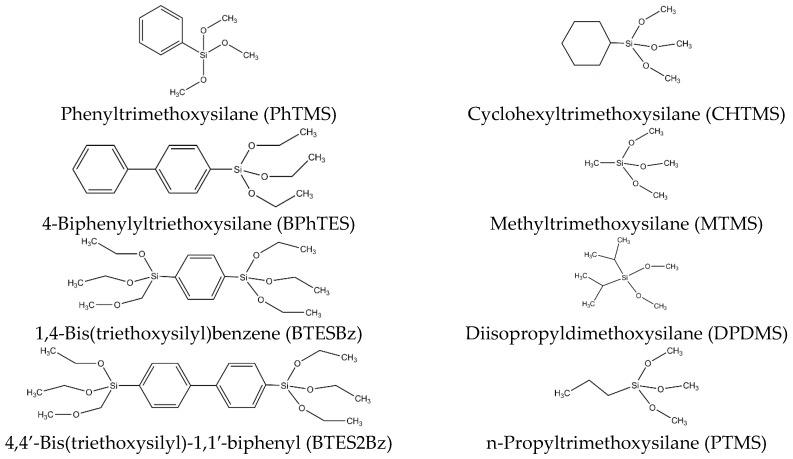
Precursors of the organosilica networks.

Organosilica materials are silicon oxide networks that contain organic groups distributed at the molecular level, i.e., a fraction of the Si–O bonds is replaced by Si–R bonds (R is alkyl, alkylene, phenyl, phenylene, etc.). The mixed organic-inorganic nature of these materials provides an interesting combination of material properties, such as the high thermal and chemical stability of silica and the hydrothermal stability and flexibility of the organic groups [1,2]. The position of the organic groups can be terminal (Si–R) or bridging (Si–R’–Si), which affects their exposure at the (internal) surface and influences the crosslink density and microporosity of the network [3]. Organosilicas can be prepared by mild and versatile sol-gel routes and applications are, for instance, molecular separation membranes [4], catalysts [5], low-k materials [6,7] and sensors [8]. Organosilica networks offer the combined advantage of a mechanically strong yet flexible network. The rigidity and strength of silica networks composed of connected silicate SiO_4_ units is supplemented by the flexibility and elasticity provided by organic segments. For example, the introduction of ethylene bridges between silicon atoms improves the fracture resistance of the material [6]; it maintains a high Young’s modulus at high porosity [6] and its flexibility allows fabrication of films with a thickness over 1 µm without crack formation during drying [9]. The addition of organics also has an influence on the thermal properties. There are reports in the literature showing the influence of certain organic moieties on the glass transition (*T*_g_) and the melting temperature (*T*_m_). For example, the use of bulky arene-terminal siloxane precursors, such as phenyltrimethoxysilane (PhTMS) yields a material with decreased *T*_g_ and *T*_m_ compared to silica. The incorporation of the bulky phenyl group results in glassy behavior upon heating, allowing re-melting of the polymer at low temperatures (<100 °C) compared to materials based on smaller monomers, such as networks derived from methyltrimethoxysilane (MTMS) [10]. Some examples of organically modified silane precursors are shown in Scheme 1.

Organosilica and silica have been investigated for their use as lubricants, which is due to their favorable chemical and structural behavior upon mechanical solicitation. When two silicon oxide surfaces come into contact in the presence of water, the rupture and reformation of siloxane bonds can lead to wear under high loads [11]. At low pH, the presence of protons (H^+^) triggers the formation of a charged hydration layer on the surfaces that reduces the reformation of the siloxane bonds and may lower the friction force [12]. The labile nature of the siloxane-silanol groups at the surface of silica seems to affect the overall friction forces generated in the contact. The lubrication mechanism of silicon oils used in metal casting is based on the sliding of the organically modified siloxane chains enhanced by low molecular attraction [13]. Silicon oil wets the surfaces and decomposes at high temperatures into silica [14]. Polysiloxanes with phenyl substituents have shown higher load capacity of the oil, a parameter related to the ability of the lubricant to prevent or reduce wear [14]. Other examples of lubricants used in high temperature processes are aqueous suspensions of graphite [15], polytetrafluoroethylene [16] and molybdenum sulfide [17]. Their lubrication mechanism is similar to that of silicon oil, involving the sliding of molecules or platelets when a shear force is applied [18]. The use of these lubricants is limited by their thermal stability, availability or resulting degradation products, which can cause dirty working environments or corrosion [19]. Organically modified silica can be stable up to 600 °C [20]. In high temperature processes, its main decomposition products are CO_2_, H_2_O and SiO_2_. These decomposition products do not participate significantly in corrosion processes and are not considered as toxic waste as long as SiO_2_ is not present in the form of nanoparticles [21]. Despite the abundant exploration of the use of polysiloxane oils in lubrication and their high potential, organosilica powders have not yet been applied in high temperature solid lubrication processes.

In this study we prepared diverse powders from organosilica precursors (see Scheme 1) by sol-gel processing. The powders were suspended in water and dried on the surface of steel, yielding a thin film of loose particles on the surface of the metal. The mechanical properties of these dried solid lubricant films, influenced by the nature of the sol-gel monomer used to prepare the material, were investigated in friction tests. The carbon content and structural characteristics of several organosilica networks were compared (1) to identify suitable low-friction solid lubricants for high temperature processes, and (2) to explain the friction properties in terms of the structural, mechanical and physicochemical properties of the materials. 

## 2. Results and Discussion

**Figure 1:**
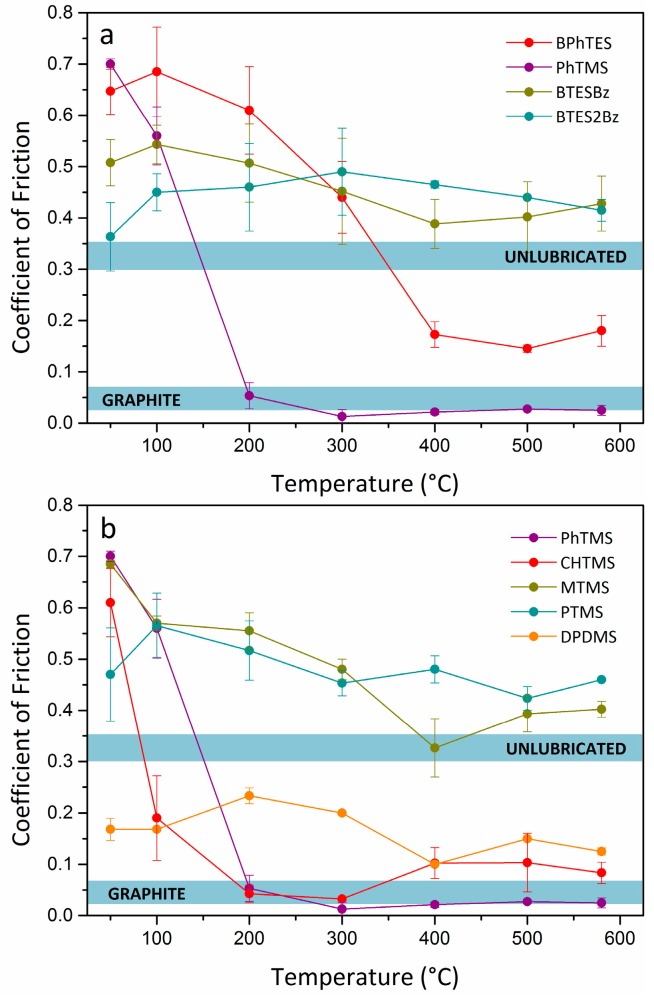
Friction coefficients of the organosilica networks. The figure displays the coefficients of friction (CoF) obtained by high temperature pin-on-disc measurements on films of organosilica powders prepared from suspensions: (**a**) Polymers obtained from monomers with terminal and bridging organic groups; (**b**) Polymers obtained from monomers with different terminal organic groups. Reference CoF of the unlubricated metal-metal contact and a graphite film are included.

The lubricative properties of different organosilica networks with terminal and bridging organic groups were studied with high-temperature pin-on-disc (PoD) experiments. The solid lubricant was a loosely bound powder film obtained from evaporation of a suspension on the flattened pin. A schematic representation of the experiment and the pin used is shown in Figure S1, in the supporting information. The supplied amount of lubricant was sufficient to realize lubrication under hydrodynamics conditions, i.e., conditions where the lubricant film thickness far exceeds the roughness of substrate and pin. However, below the softening temperature of the lubricant (see below), it is possible that a mixed lubrication or boundary lubrication mechanism may have been operative. The recorded friction forces were translated into a coefficient of friction (CoF) and these are shown in Figure 1. 

The CoF of the organosilica materials were compared with the CoF of the unlubricated metal-metal contact, and a film of the state-of-the-art lubricant graphite. For the sake of clarity and simplicity, the data points of both reference measurements are presented in the form of a colored band. The actual CoF values of the references can be found in Figure S2, in the supporting information. The morphology of unmilled PhTMS and CHTMS powders is shown in Figure S3. In order to ascertain that the CoF data were not influenced by the particle size of the lubricants, CoF experiments were performed on unmilled and dry-milled powders of 0.3 or 1.0 μm average size. An illustrative example is shown in Figure S4, where CoF data of PhTMS samples with different particle sizes versus temperature clearly show identical behavior. 

**Figure 2:**
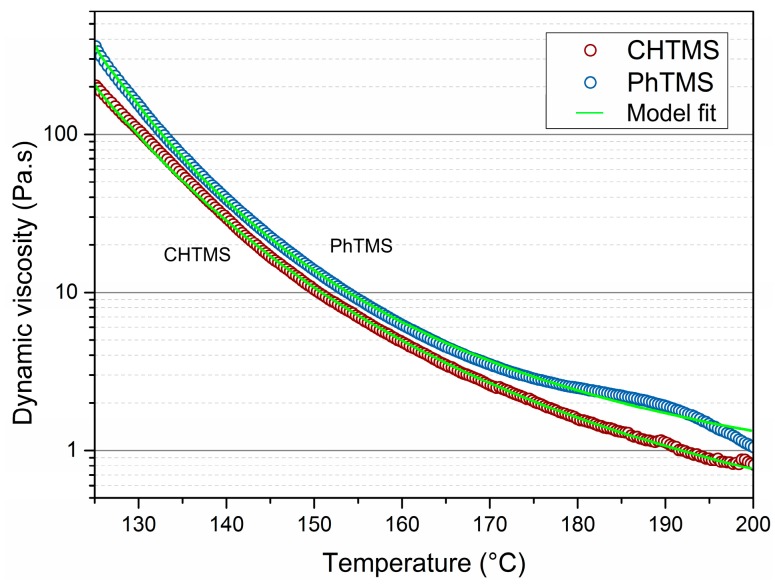
Dynamic mechanical analysis (DMA) curves of CHTMS and PhTMS polymers. The curves were recorded after the formation of a melt. Masuko-Magill model fits to the experimental data are plotted as green lines. The blue bands represent the range of values of the two references recorded at different temperatures.

Two sets of experiments were performed to relate the mechanical friction properties to (1) the structure of the monomer and (2) the carbon content, respectively. Figure 1a shows the CoFs of organosilica networks with terminal arene and bridging arylene groups. The data at 25–100 °C show a high CoF and large scatter in the experimental data. The particles triggered a slip-stick motion of the pin, typical for loose particles that roll and slide over the contact asperities. However, the polymers with terminal hydrocarbon groups, based on PhTMS or BPhTES precursors, showed a remarkable viscosity drop at higher temperatures, as discussed in more detail below (Figure 2). The CoF of PhTMS-derived material decreased abruptly from 0.7 at 50 °C to 0.01 at 300 °C with the formation of a low-viscosity phase above 100 °C. Low *T*_g_ and *T*_m_ values of 50 °C and 150 °C, respectively, have been reported in literature for PhTMS-derived material, and although the degree of polymerization in those studies may have been different than in the present study, they are in the range of temperatures where the CoF drop occurred [10,22]. The coefficient of friction recorded from the first data points was comparable to or even lower than that of the graphite reference lubricant. The softening behavior of the PhTMS-derived powder disappeared within few minutes and this happened even faster when the disc temperature was higher. The latter behavior is thought to originate from ongoing condensation of neighboring silanol groups into siloxane bonds and the gradual formation of a more rigid 3D network in the glass upon heating. More details can be found in the discussion of the Diffuse Reflectance Infrared Fourier Transform Spectroscopy (DRIFTS) spectra below. After the friction experiments, powder-like residues remained behind on the disc.

In contrast to the softening observed in materials derived from monomers with terminal organic groups, the organosilicas with bridging organic groups, i.e., BTESBz and BTES2Bz, did not show signs of softening leading to lower CoF values. They showed comparable trends and CoF values above the non-lubricated metal-metal contact. No clear correlation can be established between the gross aromatic ring content and the mechanical friction properties of BTESBz and BTES2Bz derived powders, as was found for the materials obtained from monomers with terminal organic groups. Materials with bridging arylene groups seem not to reach the degree of plastic deformability of organosilicas with terminal arene groups. The differences in mechanical behavior between organosilicas with bridging and terminal organic groups can be explained in terms of network connectivity and crosslink density differences. Silicon monomers with terminal organic groups have condensable functional alkoxy groups and can thus form up to three bonds with other monomers, while monomers with 2 Si centers and a bridging organic group can form up to six bonds per monomer. Therefore, the increased connectivity in the network probably yields stiffer polymers that do not undergo substantial softening upon heating. 

In general, the presence of bulkier organic terminal moieties in the organosilica monomers led to lower CoF values for the condensed materials, as can be seen in Figure 1b. The figure displays organosilica networks with varying carbon contents and structures. Materials obtained from monomers with low carbon content, i.e., MTMS and PTMS, showed behavior indicative of rigid materials, characterized by high CoF values at all temperatures and large scatter in the experimental data compared to the networks with higher carbon contents that were more deformable. The overall trend from low to high carbon content is an apparent flexibility increase of the network with an enhanced plastic deformability under mechanical loads. The polymer DPDMS yielded low friction coefficients from room temperature upwards. The presence of only two alkoxide groups in the DPDMS monomer yields linear polymers instead of 3D connected networks after polymerization. This network of linear chains seems to be particularly capable of absorbing the shear forces at low temperatures in a manner comparable to silicon oils, with which it is in fact structurally related. They too are linear rather than cross-linked polymers such as the other organosilicas in this study. Polymers derived from monomers with terminal organic groups and high carbon contents, i.e., PhTMS and CHTMS, yielded low CoF values at high temperatures. Both network structures, with bulky carbon rings as terminal groups, showed low friction coefficients, down to values of 0.01–0.03. Their CoF was considerably lower than that of the linear polymer. We think that the superior thermomechanical behavior of PhTMS and CHTMS-based lubricants is partly related to the cross-linked structure of these polymer networks and to the bulkiness of the hydrocarbon moieties.

The comparatively higher CoF found for BPhTES-derived material than for PhTMS-derived material may be explained by considering that the aromatic organic groups tend to interact via their delocalized electron charge which can induce π-π stacking [23]. BPhTES-derived materials may have enhanced stabilization of the polymeric network through ring stacking of the biphenyl groups of the constituent monomers, compared to PhTMS-derived materials with only one phenyl ring per monomer. As a result of these interactions, the PhTMS oligomers may have a higher mobility in the melt compared to their biphenyl counterparts, possibly resulting in a lower CoF for the PhTMS-derived material. 

The two organosilicas with lowest CoF at high temperature, i.e., PhTMS and CHTMS-derived powders, were subjected to dynamic mechanical analysis. Data are shown in Figure 2. They display the change in dynamic viscosity upon heating to 200 °C. The viscosity decreases with increasing temperature as the result of reduced friction between molecules, arising from reduced intermolecular interactions with increasing molecular free volume. It can be seen that the dynamic viscosity for both networks decreased by at least two orders of magnitude in this temperature interval. The changes are gradual, and no clear transitions can be seen in the Differential Scanning Calorimetry (DSC) data in Figure S5, in the supporting information. The lowest recorded viscosities were 0.7 Pa∙s for CHTMS, and 1.0 Pa∙s for PhTMS at 200 °C. The Masuko-Magill model was fitted to the experimental viscosity data [24]

(1) $$\log\left(\eta /\eta_{g}\right)=Ae^{B\left(T_{g}-T\right)/T}-1$$

where *η* and *η*_g_ are the viscosities at a given temperature *T* and at *T*_g_, respectively. *A* and *B* are constants depending on the individual polymers. For the model fit, *T*_g_ was estimated to coincide with the starting temperature of the experiment (125 °C). The best fits of Equation (1) to the experimental data yielded *A* = 7.71, *B* = 8.1 for CHTMS, and *A* = 6.74, *B* = 11.22 for PhTMS. The trends are consistent with the behavior of a typical polymer that gradually undergoes partial melting above the glass transition temperature. This melting may even lead to the (temporary) formation of oligomers because of depolymerisation due to hydrolysis of Si–O–Si bonds [25]. The formation of a melt upon heating explains the good lubricious performance of CHTMS- and PhTMS-derived networks. The lower viscosity of the CHTMS-derived material is consistent with its lower CoF in this temperature range, as compared to PhTMS-derived material.

**Figure 3:**
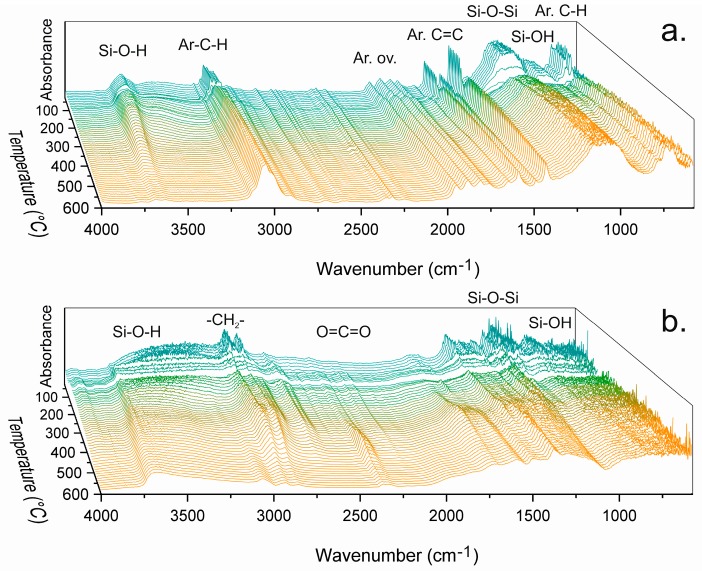
Temperature-dependent diffuse reflectance FTIR spectroscopy (DRIFTS) data of several organosilica networks. Condensed material derived from (**a**) PhTMS; (**b**) CHTMS.

The chemical changes in powders derived from PhTMS, BPhTES, BTESBz, BTES2Bz and CHTMS were monitored with DRIFTS during heating from room temperature to 600 °C. The powders were not dried prior to analysis, causing significant loss of adsorbed water and synthesis residues (ethanol, methanol) at temperatures up to approximately 150 °C. Distinguishing spectral features is hindered by the intrinsically strong absorbance of silica moieties and the disordered nature of the network. DRIFTS spectra of PhTMS and CHTMS are shown in Figure 3. They show isolated silanol O–H stretching vibrations (~3690 cm^−1^) [26], hydrogen-bonded O–H stretching vibrations (3600–3200 cm^−1^) [27], siloxane Si–O–Si stretching vibrations (1150–1000 cm^−1^) [26,27] and silanol Si–O stretching vibrations (~900 cm^−1^) [26]. The spectra of BPhTES, BTESBz and BTES2Bz can be found in Figure S6, in the supporting information. 

The aromatic moieties, phenyl(ene) and biphenyl(ene), show well-defined bands indicative of aromatic C–H stretching vibrations (3080–3010 cm^−1^), aromatic overtones and combination bands (2000–1650 cm^−1^), aromatic C=C stretching vibrations (1625–1430 cm^−1^), aromatic out-of-plane C–H deformations and out-of-plane ring vibrations (900–650 cm^−1^) [27]. The bands at 2000–1650 cm^−1^ and 900–650 cm^−1^ indicate the number of substituents on aromatic rings [28] and thus they also indicate the integrity of the Si–C bonds. A list of all indexed peaks can be found in Table S1, in the supporting information. 

The spectra of PhTMS-derived material indicate intrinsically lower amounts of water compared to other networks. The typical absorption peak of water at 1630 cm^−1^ was not present in the spectra and although the hydroxyl absorption bands from water overlap with the hydrogen-bonded silanol vibration bands in the 3600–3200 cm^−1^ region, no molecular water seemed to be present in the network. The insignificant mass loss in TGA up to 200 °C (see below) supports this observation. 

The most remarkable change in the spectra of PhTMS-derived material (Figure 3a) is the sudden intensity drop of all absorption peaks around the temperature region where softening was recorded by PoD. The intensity decay started at 105 °C until partial recovery at 145 °C and was attributed to the formation of a less IR-active state upon thermal relaxation and/or melting of the network. The absorbance changes indicate the formation of an intermediate state involving flow of material that may or may not involve chemical changes, i.e., bond breakage and reformation [29]. Solid-state silicon nuclear magnetic resonance studies have shown that PhTMS-derived material can have relatively high concentrations of silanol groups, but also that the network may undergo ongoing condensation 2 Si–OH → Si–O–Si + H_2_O with increasing temperature [10]. This suggests ongoing organization of the three-dimensional organosilica network in the flowing state and increasing condensation of the silanol groups upon further heating [29]. Indeed, the DRIFTS spectra in Figure 3a indicate ongoing condensation with increasing temperature, evidenced by the decay of hydroxyl vibrations at 3600–3200 cm^−1^ and silanol vibrations around 900 cm^−1^. The progressive condensation of silanol groups into siloxane bonds was found to be an irreversible process, as demonstrated in Figure S7 in the supporting information, and in agreement with the hardening observed for PhTMS during the CoF measurements as discussed above. The degree of condensation is directly coupled to the disappearance of viscous reflow properties in PhTMS-derived powders. Overall, PhTMS networks were found to be thermochemically stable against oxidation up to 600 °C in air under the conditions of our measurements. 

The spectra of CHTMS-derived material in Figure 3b showed behavior similar to that of PhTMS-derived material, with a large absorbance decrease between 125 and 155 °C, i.e., the same temperature range as where the CoF drop occurred. The DRIFTS spectra showed non-aromatic C–H stretching vibrations (3000–2800 cm^−1^), with a significant intensity loss above 300 °C, accompanied by upcoming vibrations in the CO_2_ region (2350–2335 cm^−1^) and the C=O region (1600–1750 cm^−1^), probably resulting from partial oxidation. The CHTMS network shows a temporary peak in the C=C stretching region (1680–1630 cm^−1^) [27] upon temperature increase, indicating the formation of intermediate degradation products that degrade further upon further heating. All these observations suggest thermal degradation of CHTMS-derived material, occurring at lower temperatures than in the corresponding PhTMS networks with aromatic terminal groups, which seems to affect the lubricative properties. Indeed, the CoF underwent a slight increase above 300 °C, at the onset of thermal degradation, as can be seen in Figure 1. The spectra of BPhTES-, BTESBz- and BTES2Bz-derived materials in Figure S6 show similar behavior in terms of thermal stability. 

**Figure 4:**
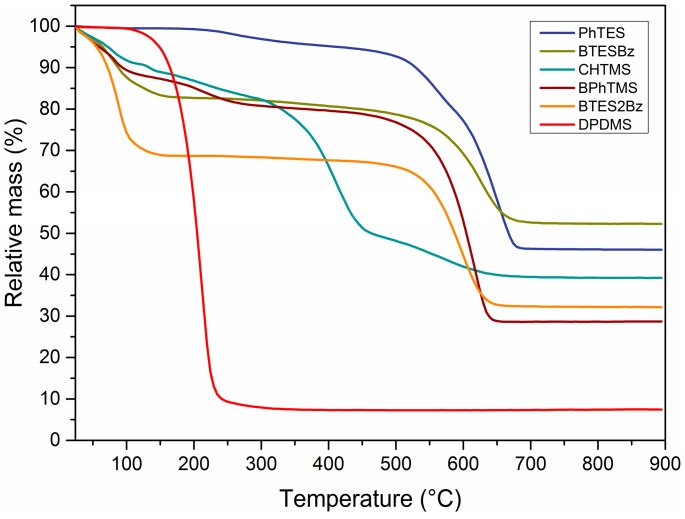
Thermal evolution of different organosilica materials. Thermogravimetric analysis (TGA) of materials derived from PhTMS, BPhTES, CHTMS, BTESBz, BTES2Bz and DPDMS in flowing air. Heating rate of 5 °C/min.

Overall, the reduction in friction coefficient of PhTMS and CHTMS above *T*_g_ and the increasing CoF of CHTMS upon thermal degradation can be correlated with the events recorded by DRIFTS. The high friction coefficients of BTESBz- and BTES2Bz-derived materials may be related to the high thermal stability as recorded by DRIFTS. Figure 4 shows thermogravimetric analyses of several organosilica networks that have been subjected to PoD experiments.

In agreement with the DRIFTS spectra, the PhTMS-, BTESBz- and BTES2Bz-derived materials are thermally stable up to 600 °C in a stagnant air environment, and their stability decreases only by 20 to 50 °C under a flow of air. In general, the mass losses under 150 °C correspond to evaporation of physisorbed water and ethanol/methanol from the pores of the network. The solvent residues did not visibly influence the lubricative properties, as seen in Figure 1 for all polymers that did not melt under 200 °C. The polymers derived from monomers containing bridging arylene groups, i.e., BTESBz and BTES2Bz, did not show further mass loss until the beginning of organic degradation at 550 °C. PhTMS-derived powders did not show a significant mass loss below 200 °C, which indicates the absence of adsorbed solvents in the powder. The mass loss starting from 208 °C can be associated with the process of condensation of residual silanol Si–OH groups into siloxane Si–O–Si bonds, with consequent water release and a quantitative loss of approximately one OH group per monomer. 

The progressive mass loss for CHTMS-derived material above 300 °C corresponds to the onset of organic decomposition as witnessed by the decay of C–H vibrations (DRIFTS) in Figure 3 and the slight increase in CoF in Figure 1. The compound experienced almost total degradation from 295 °C onwards. The DPDMS-derived network did not show any mass loss related to adsorbed water or synthesis residues. However, a mass loss of over 90% occurred above 178 °C, indicating the evaporation of DPDMS. The short-timed PoD measurements allowed, however, to record friction coefficients during the first contact between the two metal surfaces. Overall, the materials obtained from monomers containing bridging moieties, BTESBz and BTES2Bz, showed a relatively high thermal stability and a poor performance as a solid lubricant compared to materials containing terminal organic species. These latter materials showed low CoF, in some cases comparable to or lower than graphite, especially at temperatures above their apparent *T*_g_. The structure, carbon content and connectivity of the network are key factors affecting the mechanical properties of the organosilica as solid lubricants.

## 3. Materials and Methods

### 3.1. Chemicals

Methyltrimethoxysilane (97%), n-propyltrimethoxysilane (98%), diisopropyldimethoxysilane (95%), cyclohexyltrimethoxysilane (97%), phenyltrimethoxysilane (97%), 4,4′-bis(triethoxysilyl)-1,1′-biphenyl (95%) and 1,4-bis(triethoxysilyl)benzene (95%) were obtained from ABCR and 4-biphenylyltriethoxysilane (>95%) was obtained from Gelest (Morrisville, PA, USA). Nitric acid was purchased from Acros (65 wt % aqueous solution) (Geel, Belgium) and Sigma Aldrich (70 wt % aqueous solution) (Zwijndrecht, The Netherlands). Ethanol (dehydrated, 99.99%) was obtained from VWR (Amsterdam, The Netherlands). All compounds were used as received, without further purification. The molecular structure of the precursors and their abbreviated names as used in this study are shown in Scheme 1.

### 3.2. Organosilica Powder Preparation 

Sols were synthesized by adding demineralized water and aqueous HNO_3_ (65 wt %) to dry ethanol at room temperature, followed by addition of the precursor under stirring. The ratio HNO_3_:H_2_O:alkoxy was kept constant at 0.064:1.1:1.0, but the overall concentration in ethanol was varied. A detailed listing of the quantities is shown in Table 1.

The mixtures were heated to 60 °C for given periods of time, cooled to room temperature in a water bath and poured in petri dishes to dry overnight in air at room temperature. MTMS, BTESBz and BTES2Bz derived materials were dried in polystyrene dishes. The other materials were dried in aluminum dishes. All powders were stored under ambient conditions. The polymers for DMA analysis were dried overnight in an oven at 140 °C to remove any residual water.

### 3.3. Characterization

Thermogravimetric analysis and differential scanning calorimetry (TGA-DSC) was performed in Pt cups using a Netzsch STA 449 F3 (Netzsch, Selb, Germany) at a constant heating rate of 5 °C/min in technical air (N_2_/O_2_ = 80/20) and temperatures between 20 °C and 900 °C. Temperature-dependent diffuse reflectance infrared fourier transform spectroscopy (DRIFTS) was carried out with a Bruker Tensor 27 (Bruker Nederland bv, Leiderdorp, The Netherlands) equipped with a Harrick Praying Mantis set-up (Harrick Scientific, Pleasantville, NY, USA) including a reaction chamber dome enclosed by ZnSe windows. DRIFTS data were recorded in the wavenumber range 4000–500 cm^−1^ with a resolution of 1 cm^−1^. Organosilica powders were placed in the reaction chamber cup with uncompacted and non-dried KBr powder and the infrared signal of KBr was subtracted from the recorded spectra. The KBr powder was renewed for every organosilica infrared measurement; variation in the background may influence the water and hydroxyl signals. The chamber was vented to the lab atmosphere through two openings. 

The dynamic mechanical analysis (DMA) curves were measured on an ARES G2 of Waters-TA Instruments (TA Instruments, Etten-Leur, The Netherlands). The applied geometry consisted of two parallel plates with a diameter of 25 mm, placed at 1 mm separation. The samples were heated from 125 °C to 200 °C with a linear heating rate of 2 °C/min. 

The lubricious properties of the organosilica powder films were assessed by the use of the High-Temperature pin-on-disc (PoD) Tribometer (CSM instruments, Needham Heights, MA, USA). The organosilica powders were milled on a roller bench using yttria-stabilized zirconium oxide (YSZ) beads (Ø = 1 mm) (Gimex Technical Ceramics BV, Helmond, The Netherlands) for 48 h in water at a concentration of 2 wt %. The pins consisted of flattened steel bearing balls (10 mm diameter, G10T SKF, SKF, Göteborg, Sweden). The surface of the steel pin was 27 mm^2^, giving a surface concentration of lubricant of 75 μg/mm^2^ (or 75 g/m^2^). The applied load was 10 N, resulting in an effective contact pressure of 370 kPa. See Figure S1 for a schematic representation of the experimental setup. The rotating disc was made of EN 10278 steel and was polished, as well as the flattened bearing balls, with SiC paper with a maximum mesh of 2000 and diamond paste of 3 μm particle size. The calculated arithmetic roughness of the steel surfaces was *R*a = 0.90 ± 0.25 μm. The friction force was recorded for a maximum of one revolution of the disc under the pin, at a velocity of 1 mm/s. Data were collected from the first data points after contact between the pin/lubricant and the disc at a preset temperature varying between 25 and 580 °C. The coefficient of friction (CoF) was averaged over a minimum of 5 repetitions. The typical time scale of one experiment was 30 s since the powder films were susceptible to be worn out due to the limited reservoir surface of the pin. The friction force of graphite suspensions was used as a reference. The graphite powder (99.99%, Sigma-Aldrich, Zwijndrecht, The Netherlands) was milled on a roller bench with YSZ beads (Ø = 1 mm) for 48 h and applied to the steel pins in an identical way to the organosilica suspensions. 

## 4. Conclusions

The introduction of organic groups to the silicon oxide network affects the tribological properties of the lubricants. The thermal stability of the organosilica phase is enhanced by the presence of aromatic groups as compared to aliphatic ones. The mechanical friction properties seem to be controlled by the polymer network structure. Polymers obtained from 3-functional monomers with terminal organic groups showed lower CoFs above a certain threshold temperature than organosilica polymers with organic bridging group, and linear chain-like polymers. The higher crosslink density of monomers with six condensable functional groups, compared to monomers with only three condensable and one terminal organic groups, is likely to lead to less easily deformable networks. Indeed, the most easily deformable DPDMS-derived linear chain-based polymers yielded low friction forces at all temperatures. The overall picture that emerges from these experiments is that organosilicas made from 3-functional PhTMS and CHTMS form not so densely cross-linked oligomers, that undergo thermal relaxation, softening, deformation and even (partial) melting with increasing temperature. However, residual unreacted functional groups present in these oligomers undergo further condensation at higher temperatures, eventually leading to high molecular mass polymers that do not have the same lubrication capabilities as their oligomeric equivalents. The PhTMS- and CHTMS-derived networks seemed to perform best as solid lubricants, reaching CoF values of 0.01–0.03 at elevated temperatures. The other networks, in particular the 6-functional ones containing organic bridging groups, may be too highly cross-linked to be plastically deformable and potentially useful as high temperature solid lubricant.

## Figures and Tables

**Table 1:** Quantities and reaction times employed for the synthesis of the organosilica powders.

Precursor	Ethanol (cm^3^)	Water (cm^3^)	HNO_3_ 65 wt % (cm^3^)	Precursor (cm^3^)	Reaction Time (min)
MTMS	35.0	4.11	1.01	10.87	180
PTMS	60.0	2.38	0.585	7.73	50
DPDMS	70.0	1.04	0.255	5.80	180
CHTMS	70.0	1.57	0.387	5.95	50
PhTMS	80.0	1.78	0.439	6.17	50
BPhTES	27.0	0.40	0.098	2.23	110
MTMS	35.0	4.11	1.01	10.87	180
BTES2Bz	33.0	1.04	0.256	4.41	50

## Supplementary Materials

The following are available online at http://www.mdpi.com/1996-1944/11/2/180/s1, Figure S1: Schematic representation of the pin-on-disc setup and the shape of the pin, Figure S2: Variation of the average CoF with temperature of graphite and unlubricated surfaces, Figure S3: SEM images of PhTMS and CHTMS powders, Figure S4: CoF versus temperature of PhTMS samples with different grain sizes, Figure S5: DSC graphs of PhTMS and CHTMS, Temperature-dependent DRIFTS data of BPhTES, BTESBz, and BTES2Bz, Figure S6: Temperature-dependent DRIFTS spectra of PhTMS-derived material with three successive temperature cycles, Figure S7: Temperature-dependent DRIFTS spectra of PhTMS-derived material with three successive temperature cycles, Table S1: Peak list and assignations of temperature-dependent DRIFTS spectra in Figure 3.
