# Supplementary material for: Thermochemical Stability and Friction Properties of Soft Organosilica Networks for Solid Lubrication
Source: Materials (Basel). 2018 Jan 24;11(2):180. doi: 10.3390/ma11020180 (PMC5848877; doi:10.3390/ma11020180)
Supplement: Supplementary file 1 [file materials-11-00180-s001.pdf]

Supporting Information

Thermochemical stability and friction properties of soft organosilica networks for solid lubrication

Pablo Gonzalez Rodriguez,[1-2] A. Petra Dral,[2] Karin J.H. van den Nieuwenhuijzen,[2] Walter Lette,[3] Dik J. Schipper,[3] Johan E. ten Elshof[2,*]

[1] Materials innovation institute (M2i). Elektronicaweg 25, 2628 XG Delft, The Netherlands.

[2] Inorganic Materials Science, MESA+ Institute for Nanotechnology, University of Twente, P.O. Box 217, 7500 AE Enschede, The Netherlands.

[3] Surface Technology and Tribology, Faculty of Engineering Technology, University of Twente, P.O. Box 217, 7500 AE Enschede, The Netherlands.

[*]Correspondence: j.e.tenelshof@utwente.nl. Phone +31-53-489-2695.

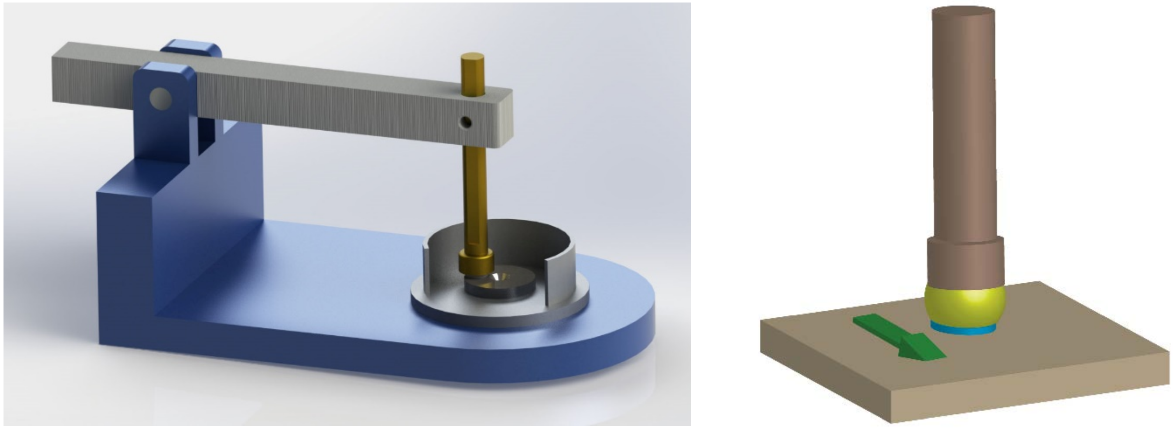

Figure S1. Schematic representation of the pin-on-disc setup and the shape of the pin used in the determination of the friction coefficient.

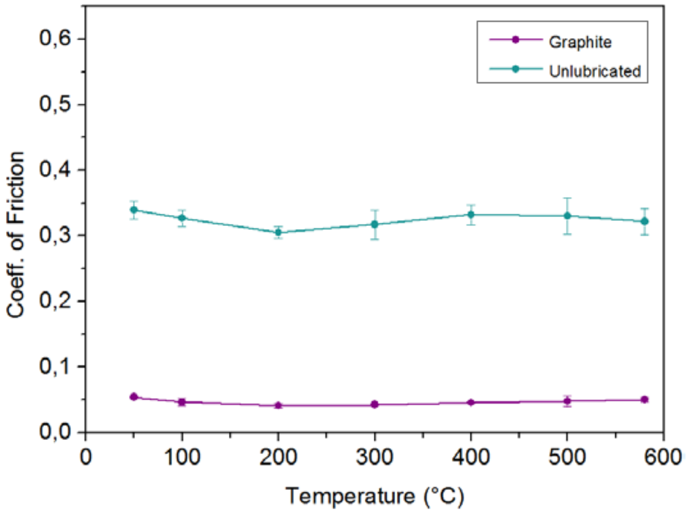

Figure S2. Variation of the average coefficient of friction with temperature of graphite and the unlubricated surfaces used as reference values in this work.

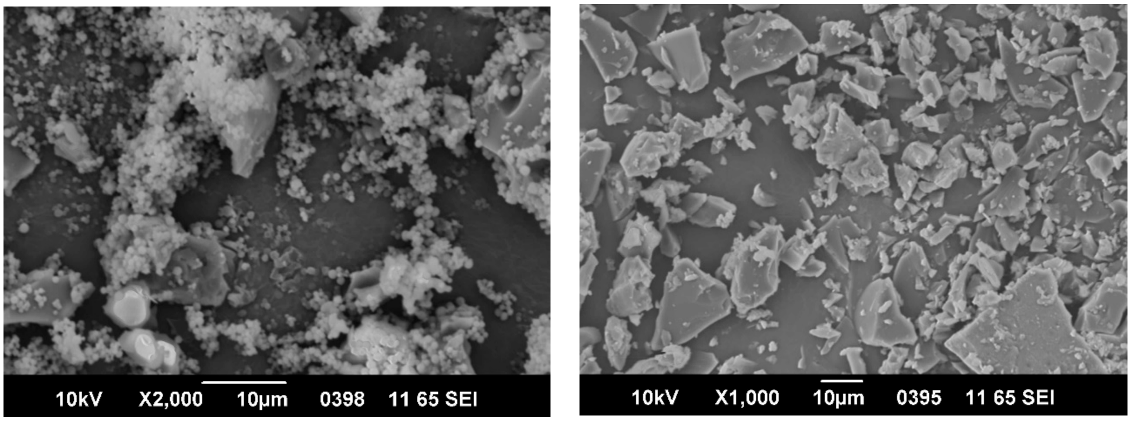

Figure S3. Secondary Electron SEM images of PhTMS and CHTMS powders, illustrating the morphology of as-made material. Prior to CoF measurements, these powders were ball-milled to a grain size of ~1 µm.

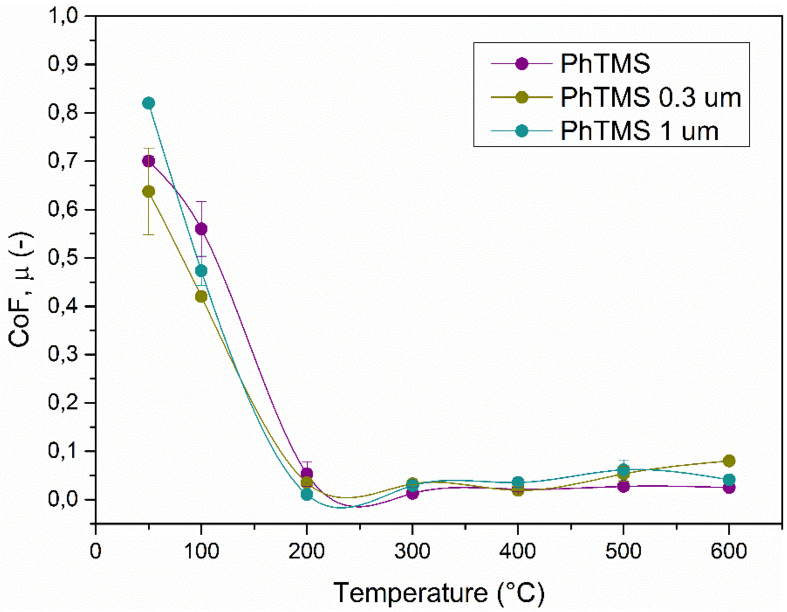

Figure S4. Comparison of the average coefficient of friction versus temperature of three PhTMS samples with different grain sizes (0.3 µm, 1.0 µm and unmilled powder). All three powders show identical behavior within experimental error.

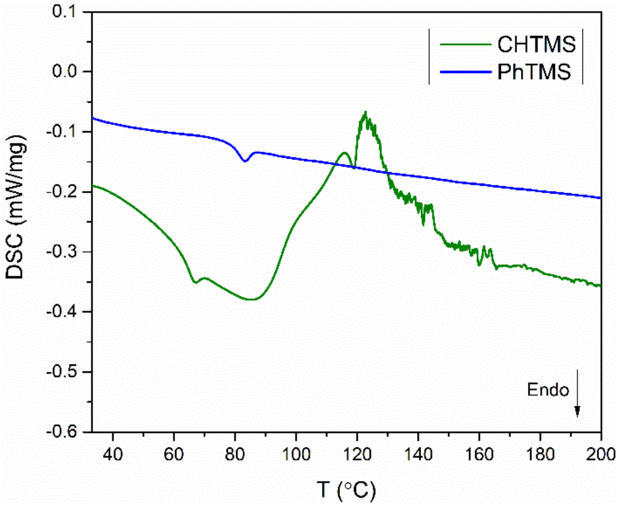

Figure S5. DSC graphs of PhTMS and CHTMS. The endothermic heat effect < 100 °C is attributed to solvent evaporation. The DSC signals above 100 °C are very small and could be attributed to several phenomena, including ongoing hydrolysis and condensation of previously unreacted group, structural rearrangements in the network, and even partial depolymerization.

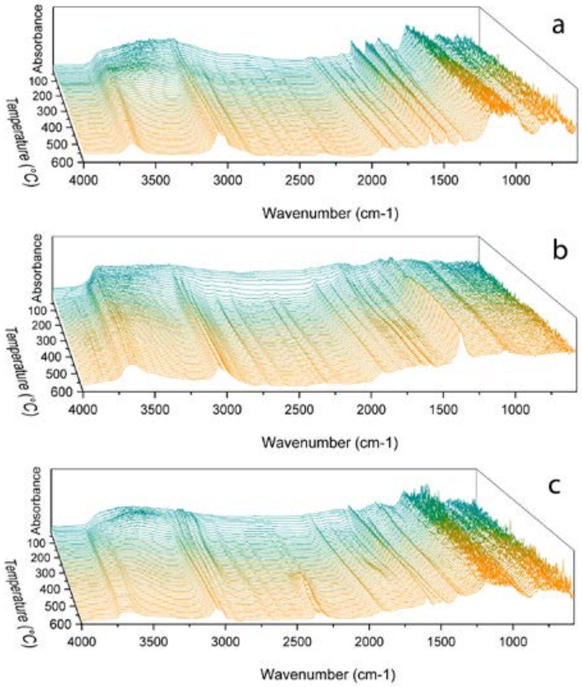

Figure S6. Temperature-dependent diffuse reflectance FTIR spectroscopy (DRIFTS) data of several organosilica networks. Condensed material derived from (**a**) BPhTES, (**b**) BTESBz, and (**c**) BTES2Bz. The spectra show upcoming peaks at the location of CO_2 vibrations (2350-2335 cm^{-1}) above 300-400 °C. This suggests some degradation of organic segments and changes in the aromatic structures (e.g. changing degree of substitution).

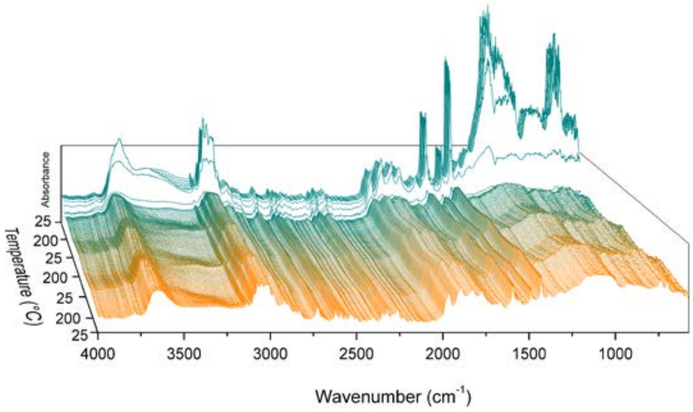

Figure S7. Temperature-dependent infrared spectroscopy (DRIFTS) of PhTMS-derived material with three successive temperature cycles 25 °C – 200 °C – 25 °C. The absorbance (vertical axis) has been scaled in order to better visualize the cycling effect on the spectra. The formation of a less infrared-active network at 105 °C upon melting was a single irreversible event and the same absorbance was not recovered upon successive cooling.

Table S1. Peak list (at room temperature) and assignations of temperature-dependent infrared spectroscopy (DRIFTS) spectra shown in Figure 3.

**Peak families**	**Materials derived from:**				
	PhTMS	BPhTES	BTESBz	BTES2Bz	CHTMS
Isolated silanol (O-H) str. vib.	3632		3600		
Hydrogen bonded (O-H) str. vib.	3456–3139	3517–3113	3508–3120	3431–3144	3332–3077
Cyclohexane (–CH_2–) str. vib.	–	–	–		2930
					2845
Aromatic (C-H) str. vib.	3073	3029	3070	3070	–
	3007	3076		2976	
	2981				
Carbon dioxide (C=O) str. vib.	–	–	2360[a]	–	2345[a]
Aromatic overtones and combination bands	1962	1924	1935	1917	1632[b]
	1892	1812	1654	1701	
	1822	1752	–	1652	
	1776	1675			
Aromatic (C=C) str. vib.	1564	1602	–	1603	–
	1490	1486		1528	
	1431	1445		1387	
Methyl (–CH_2–) in-plane def. in Si–CH=CH_2	1379	1387	1326	–	–
	1308	1337	1364		
C-H bending in O_3Si-CH_2	–	–	–	–	1276
Siloxane (Si-O-Si) str. vib.	1129–1103	1158–1020	1163–1114	1155–1073	1152–1031
Silanol (Si-O) str. vib.	909	902–832	915	930–879	901
Aromatic out-of-plane (C-H) def. vib. and ring vibrations	742–638	760–661	743–668	808–645	756–667
Unidentified vibrations (Possibly CH vibrations from impurities, degradation or solvent related)	2922	2555	–	2927	2666
	2915	2512	–	2357	1356
	2901			2322	1197
	2855				
	2725				
	2321				

[a] Appearing after 325 °C, [b] Temporarily appearing at 175 °C. Abbreviations: str. vib.: stretching vibrations, def. vib: deformation vibrations.
